# Cytokine response to pregnancy-associated recrudescence of *Plasmodium berghei* infection in mice with pre-existing immunity to malaria

**DOI:** 10.1186/1475-2875-12-387

**Published:** 2013-11-01

**Authors:** Rosette Megnekou, Trine Staalsoe, Lars Hviid

**Affiliations:** 1 Centre for Medical Parasitology, Department of Clinical Microbiology and Department of Infectious Diseases, Copenhagen University Hospital (Rigshospitalet) and Institute for International Health, Immunology, and Microbiology, CSS Building 22, Øster Farimagsgade 5, 1014 Copenhagen K, Denmark; 2 Biotechnology Centre and Faculty of Sciences, University of Yaounde I, Yaounde, Cameroon; 3 Centre for Medical Parasitology, University of Copenhagen, Building 22, Øster Farimagsgade 5, 1014 Copenhagen K, Denmark

**Keywords:** Placental malaria, Cytokines, Plasmodium berghei, Pregnancy-associated parasite recrudescence, Pathogenesis, Immunity

## Abstract

**Background:**

During childhood, residents of areas with stable transmission of *Plasmodium falciparum* parasites acquire substantial protective immunity to malaria, and adults therefore rarely experience clinical disease episodes. However, susceptibility to infection reappears in pregnant women, particularly primigravidae. This is due to appearance of antigenic parasite variants that are restricted to pregnancy. Variant-specific immunity also governs pregnancy-associated recrudescence of *Plasmodium berghei* infection in pregnant mice. Pregnancy-related changes in the plasma cytokine levels of mice with immunity acquired prior to first pregnancy have not been studied in detail previously, and were the topic of the present study.

**Methods:**

A multiplexed bead assay was used to measure plasma levels of IL-5, IL-10, IL-12, IL-13, IFN-γ and TNF in BALB/c mice immunized against *P. berghei* K173 by repeated infection and drug cure before the first pregnancy. The association between cytokine levels on the one hand and parasitaemia and haemoglobin levels on the other, in mice that had never been pregnant or were pregnant for the first, second or third time were evaluated by Mann–Whitney test and Spearman rank-order correlation analysis.

**Results:**

Pregnancy *per se* did not further increase the already high cytokine levels in mice previously immunized by repeated infection and drug cure. Levels of all the cytokines except IL-10 were correlated with each other, and with parasitaemia and haemoglobin levels. Furthermore, levels of all cytokines were positively correlated with parity, except IL-10, which was negatively correlated with parity. High levels of IL-10 and low levels of the other cytokines were associated with poor pregnancy outcome.

**Conclusions:**

High levels of IL-10 and low levels of the other cytokines were associated with poor pregnancy outcome in this mouse model of placental malaria. Since the model replicates key parasitological and immunological features of placental *P. falciparum* malaria, it underpins its usefulness in immunology and pathogenesis studies of this important cause of mother/child morbidity in endemic areas.

## Background

In areas with stable transmission of *Plasmodium falciparum* parasites, women are at high risk of developing placental malaria, despite the pre-existing protective immunity acquired prior to the first pregnancy [1]. Apart from the importance of variant-specific antibody responses [2], the consequences of malaria infection during pregnancy is undoubtedly influenced by infection- and pregnancy-induced changes in cytokine levels. Successful pregnancy is characterized by the dominance of Th2 type cytokines which down-regulate Th1 type responses that could be detrimental to the foetus [3-5], whereas Th1 cytokines have been found to protect women against placental malaria [6]. This implies that pregnancy outcome in endemic areas is determined by a precarious balance between pro-inflammatory and anti-inflammatory responses, but the literature is ambiguous about the nature of this balance [7-11].

Studies of immune responses and pathogenic mechanisms in pregnant women infected by *P. falciparum* parasites are complicated for obvious reasons, and a reliable animal model would be highly beneficial [12]. Most studies on the impact of malaria infection on pregnancy in mouse models have so far used animals without pre-existing immunity. This complicates the extrapolation of findings to a human context, since the majority of the burden of malaria in pregnancy is borne by women who are living in areas with stable transmission of parasite, and who therefore have acquired substantial immunity to malaria well before their first pregnancy [2]. However, several authors have shown that the mouse model developed in the 1980s by Eling and collaborators to study the impact of pregnancy on immunity to *P. berghei* infection [13-15], shares many immunological and pathogenic aspects with placental malaria in women [16,17]. On this basis, cytokine responses in this model and their relation to pregnancy outcome were investigated.

## Methods

### Mice

BALB/c mice were purchased from Taconic (Lille Skensved, Denmark). The animals were maintained on a 12 h/12 h dark/light cycle with food and water *ad libitum* at the Department of Experimental Medicine, University of Copenhagen, Copenhagen, Denmark, in accordance with the institutional Danish and European guidelines for animal experimentation and welfare. All mice used were specific pathogen-free. The Danish Animal Experiments Inspectorate (Dyreforsøgstilsynet) approved all experiments reported in this study (permission code no 2006/561-1093), as required under Danish law.

### Parasites and infections

*Plasmodium berghei* strain K173 parasites [18], kindly donated by Wijnand Eling, were used for all experiments. The parasites were maintained by weekly passage in the blood of non-immunized mice. Infections were initiated by the intraperitoneal injection of 1 × 10^6^ infected erythrocytes (IEs) in 200 μL of normal saline, and parasitaemia was monitored from the third day of infection by microscopic examination of thin, Giemsa-stained blood smears obtained from tail nicks. This blood was used to determine haemoglobin levels in a Hemocue 201 instrument (Hemocue, Denmark). Mice with fulminant parasitaemia or severe clinical symptoms were killed as required under Danish law.

### Immunization

A modification of the immunization protocol described by Eling and Jerusalem [18] was used. Briefly, six- to eight-week-old mice were infected as described above. The infection was suppressed by adding 15 mg/l sulphadiazine (Sigma-Aldrich) to the drinking water from day 4 (D4) to D11 and D18 to D25 following infection. On D32, the mice were challenged using the same inoculum and route used for immunization. Mice showing very low or microscopically undetectable parasitaemia after one week were considered immune.

### Mating and pregnancy monitoring

Weights and peripheral blood parasitaemia of females to be mated were recorded prior to mating. On the following day (D0), they were put together with males (two to three females and one male per cage) for four days. The animals were not disturbed during this period to minimize stress-induced early pregnancy failure. The females were weighed when the males were removed on D4 and then left undisturbed until D10. Increase in body weight from D4 to D10 was used as evidence of pregnancy. Subsequent abrupt weight loss was taken as an indicator of pregnancy interruption. Parasitaemia and body weights of the animals were monitored daily from D10. Although parasite recrudescence often occurred spontaneously in pregnant mice, they were generally re-infected on D11 to D12 (with 0.2 × 10^7^ to 1 × 10^7^ IEs from pregnant mice) to increase the frequency of recrudescence in immune mice. Some mice were mated three to four times to generate a panel of plasma samples from multigravidae.

### Collection of plasma samples

Eye blood samples were collected in capillary tubes at 08:00–10.30 on D14-18 (D13 if parasitaemia was high) from immune, non-immune pregnant and non-pregnant mice. Plasma was collected and kept at -80°C until measurement of cytokine levels.

### Measurement of cytokines

Plasma levels of cytokines were measured using the pre-mixed Procarta Cytokine Assay Kit (Procarta, Denmark) according to the manufacturer’s protocol. Briefly, 25 μL of plasma and standards were incubated with pre-mixed beads coated with antibodies specific for mouse IL-5, IL-10, IL-12/p70, IL-13, IFN-γ, and TNF in pre-wetted 96-well filter plates. After washing, plates were incubated with the detection antibody and the reaction revealed with streptavidin-phycoerythrin. The beads were analysed on a Luminex 200 IS system (Bio-Rad, Denmark). The sensitivity of the assay for each cytokine was: 1.22 pg/mL (IL-5), 2.9 pg/mL (IL-10), 4.24 pg/mL (IL-12/p70), 3.57 pg/mL (IL-13), 2.38 pg/mL (IFN-γ), and 1.3 pg/mL (TNF).

### Statistical analyses

SigmaStat (SPSS Inc, Chicago, IL, USA), and CIA see [19] software packages were used for the statistical analyses. Results were reported as means or medians with corresponding 95% confidence intervals. The Spearman rank-order coefficient (*r*_*s*_) was used to evaluate parameter association. Differences with *P* values of < 0.05 were considered statistically significant.

## Results

### The impact of immunization and pregnancy on cytokine levels

*Plasmodium berghei* infections in BALB/c mice cause a marked increase in plasma levels of many cytokines, including those measured in the present study. In agreement with this, immunization by repeated infection and sub-curative treatment led to high levels of IL-5, IL-10, IL-12, IL-13, IFN-γ, and TNF compared to uninfected mice, where levels were very low or undetectable. Comparison of cytokine levels in plasma of immunized mice that either had never been pregnant (nulligravid) or were pregnant for the first time (primigravid) did not yield any significant differences (Table 1). Levels of IL-5, IL-12, IL-13, IFN-γ, and TNF were significantly correlated (r_s_: 0.41-0.90, P(r_s_) <0.001 in all cases), whereas IL-10 levels did not correlate significantly with any of the other cytokines (r_s_ range: 0.01-0.16, P(r_s_) >0.14 in all cases). Thus, cytokine levels were markedly affected by immune status (probably as a result of the parasite exposure during immunization), whereas the impact of pregnancy on cytokine levels was minimal. The results furthermore indicate that the regulation of IL-10 differs from that of the other cytokines studied.

**Table 1:** Medians [95% confidence intervals] of plasma cytokine levels and their differences in immunized mice before and during first pregnancy

**Cytokine**	**Nulligravidae (N = 34)**	**Primigravidae (N = 84)**	**Difference**	**P(T)**^**1**^
IL-5 (pg/mL)	695 [423–942]	726 [548–877]	13 [-250–217]	0.9
IL-10 (pg/mL)	61 [46–85]	72 [61–91]	-20 [-48–0]	0.06
IL-12 (ng/mL)	13.0 [7.9–20.6]	17.0 [10.0–21.2]	0.2 [-2.9–4.3]	0.9
IL-13 (pg/mL)	19.9 [0–66.7]	7.8 [0–59.6]	0 [0–14.6]	0.6
IFN-γ (ng/mL)	8.5 [5.5–9.8]	8.8 [7.2–9.5]	0.2 [-0.9–1.5]	0.8
TNF (pg/mL)	22.3 [0.3–68.0]	20.0 [7.3–36.6]	0 [-8.4–15.8]	0.9

^1^The statistical significance of the difference between nulligravid and primigravid mice in median cytokine levels.

### Cytokine correlations with parasitaemia and haemoglobin in primigravid mice

**Figure 1:**
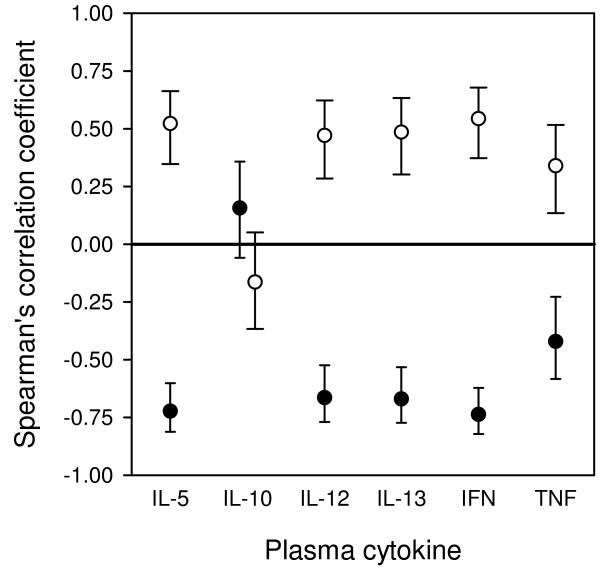
**Parasitaemia and haemoglobin and plasma cytokine levels in immunized primigravid mice.** The correlations between parasitaemia (●) and haemoglobin levels (○) and plasma cytokines were calculated. The Spearman’s correlation coefficient (r_s_) and corresponding 95% confidence intervals for each cytokine are shown.

**Figure 2:**
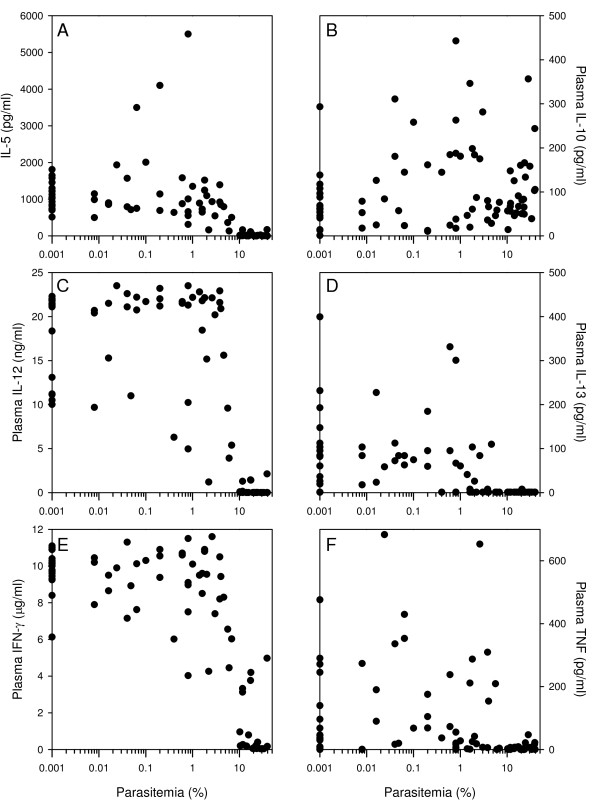
**Parasitaemia and cytokine levels in individual immunized primigravid mice.** The association between parasitaemia and plasma levels of IL-5 **(A)**, IL-10 **(B)**, IL-12 **(C)**, IL-13 **(D)**, IFN-γ **(E)**, and TNF **(F)**. Data points from individual mice are shown.

In agreement with a previous report [16], levels of parasitaemia in the present study were very low (<0.01%) in all immunized, nulligravid mice at the time of blood sampling, and their haemoglobin levels were uniformly high (median: 15.8 g/dL, 95% confidence interval: [14.8-16.1 g/dL]). In contrast, levels of both parasitaemia and haemoglobin varied widely among the primigravid mice [16]. Therefore, and because anaemia in *P. berghei*-infected BALB/c mice has been associated with high parasitaemia [20], it was examined whether cytokine levels varied with either parasitaemia or haemoglobin levels among primigravid mice. There was a significantly negative correlation between parasitaemia and all cytokines except IL-10 (Figures 1 and 2B). While levels of IL-5, IL-13, and TNF declined with increasing parasitaemia in a fairly gradual manner (Figure 2A, D, F), the relationship between IL-12 and IFN-γ on the one hand and parasitaemia on the other was essentially dichotomous (Figure 2C and E). For these latter two cytokines, high levels could be detected in most animals with less than about 10% parasitaemia, whereas all animals with higher level of parasitaemia had very low levels.

**Figure 3:**
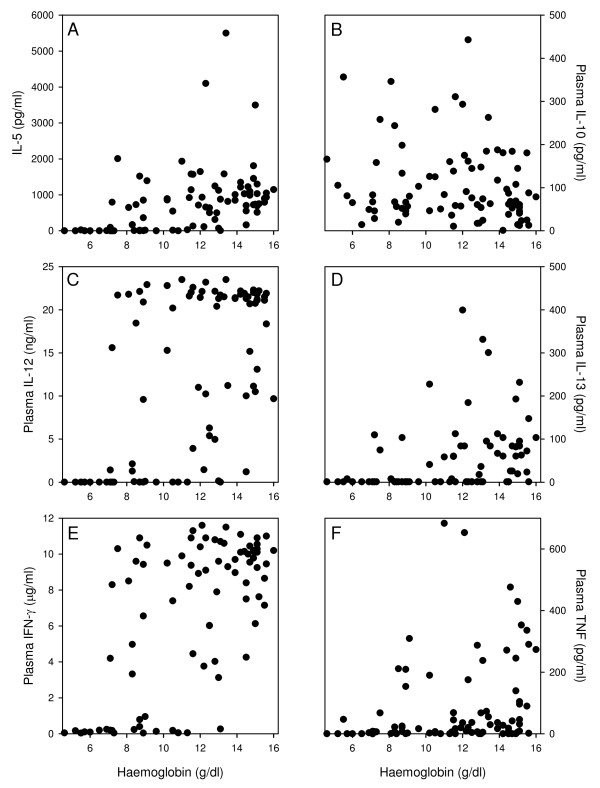
**Haemoglobin and cytokine levels in individual immunized primigravid mice.** The association between haemoglobin and plasma levels of IL-5 **(A)**, IL-10 **(B)**, IL-12 **(C)**, IL-13 **(D)**, IFN-γ **(E)**, and TNF **(F)**. Data points from individual mice are shown.

Since there was a strong negative correlation between parasitaemia and haemoglobin (r_s_ = -0.76, p <0.001), haemoglobin levels correlated significantly with levels of all cytokines (r_s_ ≥0.5 and P <0.001) except IL-10 (r_s_ = -0.18, P = 0.16) (Figures 1 and 3B). The relationship was fairly linear for IL-5, IL-13, and TNF (Figure 3A, D, F), while the dichotomous relation between IL-12 or IFN-γ and parasitaemia reported above was also observed with respect to haemoglobin levels (Figure 3C and E). Thus, animals with high levels of IL-12 and IFN-γ were never severely anaemic, while all severely anaemic animals had very low levels of these cytokines, as did a few animals with little or no anaemia.

The results indicate that the capacity to produce cytokines other than IL-10 is increasingly compromised as parasitaemia increases in primigravid mice, and that it collapses at levels of parasitaemia >10%.

### Plasma cytokine concentrations in *Plasmodium berghei*-infected mice vary with parity

**Figure 4:**
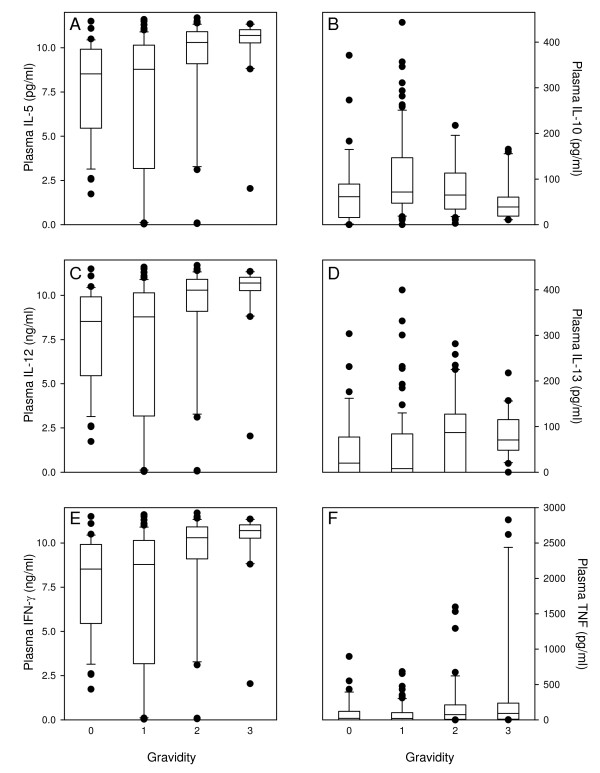
**The relationship between parity and cytokine levels in immunized mice.** Plasma levels of IL-5 **(A)**, IL-10 **(B)**, IL-12 **(C)**, IL-13 **(D)**, IFN-γ **(E)**, and TNF **(F)** among mice during their first, second or third pregnancy. Medians (horizontal line), central 50% (boxes), central 80% (whiskers) and individual outliers (●) are shown.

It has previously been reported that susceptibility to pregnancy-induced recrudescence of *P. berghei* parasitaemia and the ensuing anaemia in immunized mice decreases with increasing parity [16]. Furthermore, parasitaemia and haemoglobin levels and plasma cytokine levels were found to be related. On this basis, the relationship between parity and cytokine responses in immunized mice was examined. Levels of all cytokines were significantly associated with parity (Figure 4). Plasma levels of IL-5, IL-12, IL-13, IFN-γ, and TNF all increased (r_s_ ≥0.25, P ≤ 0.002) with increasing parity [16], whereas levels of IL-10 decreased (r_s_ = -0.25, P = 0.004), despite the lack of statistically significant relationship between IL-10 levels and parasitaemia (Figure 1).

### Plasma cytokine concentrations in *Plasmodium berghei*-infected mice vary with parity

Cytokine levels in pre-term (PTD) and full-term (FTD) deliveries were compared to determine whether there was any relationship between changes in cytokine levels and pregnancy outcome in immunized primigravid mice (see Additional file 1). PTD was defined as sudden weight loss D15-D17 in pregnant mice, whether dead pups were found or not. Significantly more (31%; 95% confidence interval [13% to 43%]) PTD than FTD mice were parasitaemic (P < 0.002), and the parasitaemia was significantly higher (P < 0.001). Levels of IL-5, IL-12, IL-13, IFN-γ, and TNF were significantly lower in PTD mice than in FTD mice, whether all the FTD mice or only the parasitaemic FTD mice were considered (see Additional file 1). Levels of IL-10 did not differ between PTD and FTD mice, but were significantly higher in parasitaemic mice than in mice without detectable parasitaemia. Thus, high levels of IL-10 and low levels of the other cytokines were significantly associated with poor pregnancy outcome (r_s_ = 0.6, P = <0.001).

## Discussion

Experimental *P. berghei* infections are uniformly lethal in mice. Death occurs either during the acute phase with symptoms of neurological dysfunction (experimental cerebral malaria) or later as a consequence of severe anaemia and hyper-parasitaemia. Mortality among *P. berghei* K173-infected BALB/c mice used here usually occurs during the chronic phase of the infection [20]. There is a broad consensus that naturally acquired immunity to malaria is mediated by both the cell-mediated and antibody-dependent mechanisms [21]. Although innate immunity plays a crucial role in clearing malaria parasites, cytokines produced by T cells are very important in enhancing and activating both innate and adaptive immune responses.

In the current study, it was found that infection of BALB/c mice with *P. berghei* K173 caused marked increases in plasma levels of pro- and anti-inflammatory cytokines including IL-5, IL-10, IL-12, IL-13, IFN - γ, and TNF. Control of malaria infection in humans need both Th1 and Th2 cytokines [22,23], and survival of BALB/c from severe inflammatory immune response due to *P. berghei* infection involves up-regulation of both pro- and anti-inflammatory cytokines [20]. It was furthermore found that plasma levels of these cytokines, except IL-10, correlated with parasitaemia, and that high levels of IL-12 and IFN-γ were associated with low parasitaemia. IFN-γ and IL-12 responses have previously been associated with protection in human malaria [11,24,25]. IL-10 levels showed a weak negative (but insignificant) correlation with haemoglobin levels. Inadequate IL-10 levels have been implicated in the pathogenesis of severe anaemia [26] and levels of IL-10 were found to be lower in placentas from *P. falciparum*-exposed women than from unexposed women [7]. Overall, the results reported here indicate that *P. berghei* K173 infection of BALB/c mice induces responses similar to those observed in human malaria infection. While most other reports regarding *P. berghei*-infected pregnant mice have used *P. berghei* ANKA, available evidence suggests that different strains of *P. berghei* behave similarly in pregnant mice, although *P. berghei* ANKA induces the strongest placental inflammation [27]. We used *P. berghei* K173 here because the recurrent recrudescences that occur in mice following immunization by drug-suppressed *P. berghei* ANKA infection are not seen after similar immunization with *P. falciparum* K173. This makes it easier to interpret recrudescences of *P. berghei* K173 following mating as being precipitated by pregnancy rather than being “random” recrudescences.

Acceptance of the foetal allograft depends on a modulation of maternal immunity toward production of Th2 cytokines [4,28], whereas protective immune responses to some parasitic infections, including malaria, involve Th1 cytokines. Thus, pregnancy outcome in parasite-exposed females is likely to be influenced by the balance between opposing responses to protect the foetus from rejection and to protect the mother from the parasites [29,30]. Results from the present study showed that levels of IL-5, IL-12, IL-13, IFN-γ, and TNF all increased with parity, which may be significant since immunity to *P. falciparum* malaria during pregnancy increases with parity [31].

Plasma levels of IL-10 were negatively correlated with parity and positively with poor pregnancy outcome (high parasitaemia, severe anaemia, abortion, and pre-term delivery) in the mouse model used here. These results agree with previous findings from studies of *P. falciparum* malaria in pregnant women [7]. Thus, elevated IL-10 plasma levels and low TNF/IL-10 ratios were associated with pre-term deliveries in Cameroon (PTDs) [9], while levels of IL-10 were elevated during placental malaria in Tanzanian and Nigerian women [32,33]. Furthermore, high IL-10 levels predicted *P. falciparum* infection during pregnancy in Tanzanian women [34], and increased IL-10 levels were associated with asymptomatic malaria in pregnant women from Ghana [35].

## Conclusions

Mice with high levels of IL-5, IL-12p/70, IL-13 and IFN-γ never were severely anaemic or had high levels of parasitaemia, while all severely anaemic mice with high parasitaemia and all PTD mice had very low levels of these cytokines. Taken together, the results suggest that this mouse model of placental malaria can be useful in studies of the mechanisms of immunity to placental malaria [2]. Carlos Penha-Goncalves and colleagues have repeatedly reported that many aspects of the pathogenesis of *P. berghei* malaria in pregnant mice resemble those seen in *P. falciparum*-infected pregnant women from endemic areas [17,36,37], reinforcing this conclusion.

## Abbreviations

IFN-γ: Interferon- γ; TNF: Tumour necrosis factor; IL: Interleukin; PTD: Pre-term delivery; FTD: Full-term delivery.

## Competing interests

The authors declare that they have no competing interests.

## Authors’ contributions

RM planned and conducted all the experimental work, wrote the draft manuscript, and participated in preparing the final manuscript. TS participated in the planning and execution of the experimental work and in preparing the final manuscript. LH participated in the planning of the experimental work and wrote the final manuscript. All authors read and approved the final manuscript.

## Supplementary Material

Additional file 1 Median levels [95% confidence intervals] of plasma cytokines in immunized primigravid pre-term delivery (PTD) and full-term delivery (FTD) mice. Click here for file
