# Supplementary material for: Cytokine response to pregnancy-associated recrudescence of Plasmodium berghei infection in mice with pre-existing immunity to malaria
Source: Malar J. 2013 Nov 1;12:387. doi: 10.1186/1475-2875-12-387 (PMC4228397; doi:10.1186/1475-2875-12-387)
Supplement: Additional file 1 — Median levels [95% confidence intervals] of plasma cytokines in immunized primigravid pre-term delivery (PTD) and full-term delivery (FTD) mice. [file 1475-2875-12-387-S1.docx]

## Additional file

## Median levels [95% confidence intervals] of plasma cytokines in immunized primigravid pre-term delivery (PTD) and full-term delivery (FTD) mice

|  | Mouse group ^1^ | | | | Statistical significance ^2^ | | |
| --- | --- | --- | --- | --- | --- | --- | --- |
| Cytokine | PTD (N=22) | FTD_All_^3^ (N=62) | FTD_Pos_ (N=43) | FTD_Neg_ (N=19) | FTD_All_ | FTD_Pos_ | FTD_Neg_ |
| IL­‑5 (pg/mL) | 2.8 [0.0-24.1] | 895 [752-1,051] | 891 [727-1148] | 900 [716-1,051] | <0.001 | <0.001 | 0.70 |
| IL‑10 (pg/mL) | 82.2 [52.0-133.5] | 68.7 [57.2-96.5] | 83.9 [60.8-161.3] | 57.2 [24.9-69.2] | 0.60 | 0.60 | 0.012 |
| IL‑12 (ng/mL) | 4.6 [0.0-11.8] | 21.3 [20.2-21.6] | 21.3 [20.2-21.8] | 21.1 [11.1-21.8] | <0.001 | <0.001 | 0.44 |
| IL‑13 (pg/mL) | 0.0 [0.0-0.0] | 60.4 [19.3-84.0] | 58.5 [0.0-74.6] | 84.0 [19.3-95.1] | <0.001 | <0.001 | 0.27 |
| IFN‑γ (ng/mL) | 0.2 [0.1-0.4] | 9.6 [9.1-10.1] | 9.5 [8.4-10.1] | 10.0 [8.9-10.3] | <0.001 | <0.001 | 0.62 |
| TNF (pg/mL) | 2.9 [0.0-7.8] | 39.1 [19.7-89.7] | 41.6 [16.4-104.4] | 31.2 [6.4-23.7] | <0.001 | <0.001 | 0.80 |

^1^PTD group includes four mice with abortion. Numbers in parentheses for each group indicate the number of mice assayed in that group

^2^Mann-Whitney signed-rank test (PTD group *vs* indicated group)

^3^Subscripts for FTD mice indicate whether all mice (All), only parasitaemic mice (Pos) or only mice without detectable parasitaemia (Neg) were included
